# Anti-HIV-1 Response Elicited in Rabbits by Anti-Idiotype Monoclonal Antibodies Mimicking the CD4-Binding Site

**DOI:** 10.1371/journal.pone.0003423

**Published:** 2008-10-16

**Authors:** Roberto Burioni, Nicasio Mancini, Donata De Marco, Nicola Clementi, Mario Perotti, Giovanni Nitti, Monica Sassi, Filippo Canducci, Krisha Shvela, Patrizia Bagnarelli, John R. Mascola, Massimo Clementi

**Affiliations:** 1 Laboratorio di Microbiologia e Virologia, Università Vita-Salute San Raffaele, Istituto Scientifico San Raffaele, Diagnostica e Ricerca San Raffaele, Milano, Italia; 2 Vaccine Research Center, National Institute of Allergy and Infectious Diseases, National Institutes of Health, Bethesda, Maryland, United States of America; 3 Laboratorio di Virologia, Università Politecnica delle Marche, Ancona, Italia; University of California San Francisco, United States of America

## Abstract

Antibodies against conserved epitopes on HIV-1 envelope glycoproteins (Env), such as the gp120 CD4-binding site (CD4bs), could contribute to protection against HIV-1. Env-based immunogens inducing such a response could be a major component of future anti-HIV-1 strategies. In this proof-of-concept study we describe the generation of two anti-idiotype (AI) murine antibodies mimicking the CD4bs epitope. Sera were collected from long-term non-progressor patients to obtain CD4bs-directed IgG, through sequential purification steps. The purified IgG were then used as Fab fragments to immunize mice for hybridoma generation. Two hybridomas (P1 and P2), reacting only against the CD4bs-directed IgG, were identified and characterized. The P1 and P2 antibodies were shown to recognize the idiotype of the broadly neutralizing anti-CD4bs human mAb b12. Both P1 and P2 Fabs were able to induce a strong anti-gp120 response in rabbits. Moreover, the rabbits' sera were shown to neutralize two sensitive tier 1 strains of HIV-1 in an Env-pseudotype neutralization assay. In particular, 3/5 rabbits in the P1 group and 1/5 in the P2 group showed greater than 80% neutralizing activity against the HXB2 pseudovirus. Two rabbits also neutralized the pseudovirus HIV-MN. Overall, these data describe the first anti-idiotypic vaccine approach performed to generate antibodies to the CD4bs of the HIV-1 gp120. Although future studies will be necessary to improve strength and breadth of the elicited neutralizing response, this proof-of-concept study documents that immunogens designed on the idiotype of broadly neutralizing Abs are feasible and could help in the design of future anti-HIV strategies.

## Introduction

Antibody-mediated neutralization is the mainstay of an effective vaccine-induced protection from viral infections [1], [2]. This theoretical starting point is deeply challenged in the case of persistent infections, as that caused by human immunodeficiency type 1 virus (HIV-1) [3]–[5]. Indeed, unlike acutely infecting viruses, HIV-1 is able to cope continuously with the human immune system, thus successfully evading the antibody response. The main targets of the anti-HIV-1 humoral response are the viral highly variable surface glycoproteins, gp120 and gp41. It is not a case that these proteins have represented the core of most anti-HIV-1 vaccines [6], [7]. However all vaccines tested to date have not been capable of stimulating a broadly neutralizing immune response, the induced protection being limited only to a minor subset of neutralization-sensitive HIV-1 isolates [6]–[11]. Indeed, these approaches have suffered from the same limitations that do not allow the mounting of an effective antibody response during the course of natural infection, that is mainly the hypervariability of HIV-1 immunodominant epitopes not fundamental for the viral biology. Moreover, another important escape strategy of HIV-1 is its ability to shield from the immune system several crucial regions of its surface antigens, such as the region of gp120 capable of binding the cellular receptor CD4 (*CD4-binding site* – CD4bs) [12]–[15]. The high protective potential of the CD4bs and of other functionally important conserved epitopes on HIV-1/gp120 has been recently highlighted in slow progressor and long-term non-progressor (LTNP) patients [16]–[18]. Indeed, several studies have shown the protective role of broadly neutralizing antibody subpopulations directed against epitopes present either on the weakly immunogenic carbohydrate loaded “silent face” [16], or on the CD4bs [17], [18]. Overall, these findings suggest that the antibody response to these epitopes could ultimately contribute, together with T-cell response, to the efficient HIV-1 infection control observed in LTNPs. The design of immunogens based on these conserved epitopes, and capable of eliciting such a protective response, could therefore be central to plan future prevention and treatment strategies of HIV-1 infection [10], [18]–[20]. Several attempts are being made to design immunogens capable of presenting to the immune systems these conserved epitopes. As far as the CD4bs is concerned, all approaches using the whole gp120 protein have been of limited success due to the high flexibility of native gp120. Indeed, gp120 can assume many decoy structures, all recognized by the immune system, but all not exposing the CD4bs and therefore stimulating antibodies irrelevant for virus neutralization [21]–[24]. An additional possibility is to use chemically modified gp120 locked in the conformation assumed by the protein when it binds the CD4, and therefore when it exposes the CD4bs [25], [26]. This approach has recently been demonstrated to be superior in the capacity of eliciting neutralizing antibodies to that based on the use of gp120 in its native form [25]. Another rational approach is based on studying the characteristics of the rare antibodies directed against the CD4bs, thus backtracking to their corresponding epitopes. The best characterized and the most potent anti-CD4bs monoclonal antibody is b12 [27]–[30], whose crystal structure in its bound form to gp120 has been recently defined [19]. Moreover, it has been recently also evidenced that b12-like CD4bs-directed neutralizing antibodies are produced in the course of the natural infection by selected *slow non-progressor* patients [18]. The idiotype of these b12-like antibodies could then be used to obtain molecular mimotopes (linear peptides, conformational disulphide-linked peptides, anti-idiotype antibodies), possibly representing the internal image of the CD4bs. Several CD4bs-mimicking peptides have been reported to date, but none of them has been able to stimulate a strong anti-gp120 neutralizing antibody response in different animal models [31]–[33]. Only in one case the immunization of rhesus macaques with a pool of phage-displayed peptides mimicking conserved HIV-1 epitopes (among which also the CD4bs) elicited a weak neutralizing antibody response against a simian isolate (SHIV-89.6PD) [34], [35]. The use of epitope-mimicking peptides is based on the assumption that the closer a peptide mimics the three-dimensional structure of an epitope, the greater is the probability that it could induce antibodies cross-reacting with the original epitope. Notwithstanding, it is particularly difficult to obtain such peptides when discontinuous conformational epitopes, as that recognized by b12 on gp120, have to be mimicked [32]. A possible solution to this problem could be to obtain conformational mimotopes, as antibodies recognizing the idiotype of other antibodies endowed with neutralizing activity against the pathogen of interest [36].

Under this perspective, in this preliminary proof-of-concept study we describe the generation of two anti-idiotype (AI) murine antibodies recognizing human polyclonal anti-CD4bs IgG, purified from LTNP. The selected AI molecules, putatively mimicking the CD4bs, were shown to be specifically recognized by b12 and to be capable of eliciting in rabbits a weak, though detectable, neutralizing response against two tier 1 HIV pseudoviruses [37].

## Results and Discussion

Sequential serum samples from 4 LTNP patients were collected over a period of 3 years; the LTNP shared the same clinical and immunological profile, including HIV-1 seropositivity for at least 8 years, CD4 T-cell counts >500 cells/µl, absence of AIDS-related opportunistic infections during the preceding 5 years, and serum neutralizing activity against at least 2 different tier 1 isolates belonging to different clades. The sera were pooled and the IgG component was purified using a protein G-containing sepharose column. Subsequently, the gp120 IIIB-binding fraction was eluted using a method described elsewhere, with minimal modification [38]. The cross-reactive CD4bs-directed antibodies were obtained by saturating the column with soluble CD4, and passing the flow-through, presumably enriched in CD4bs-directed antibodies, on a second column containing gp120 MN. Fab fragments were obtained from the anti-CD4bs IgG fraction using a commercial kit, and subsequently used for immunization of Balb/c mice for hybridoma generation. To verify the presence of possible AI monoclonals directed against the anti-CD4bs fraction, the supernatants of all hybridomas were screened in ELISA against IgG-derived Fab preparations obtained from the sera of HIV-1-negative patients. All hybridomas reacting against these control Fab preparations were not considered further; whereas non-reacting hybridomas were subsequently tested for reactivity on Fab fragments derived from anti-CD4bs IgG. After this preliminary screening, only two clones (P1 and P2) reacting specifically against the anti-CD4bs fraction were taken into consideration for subsequent testing.

**Figure 1:**
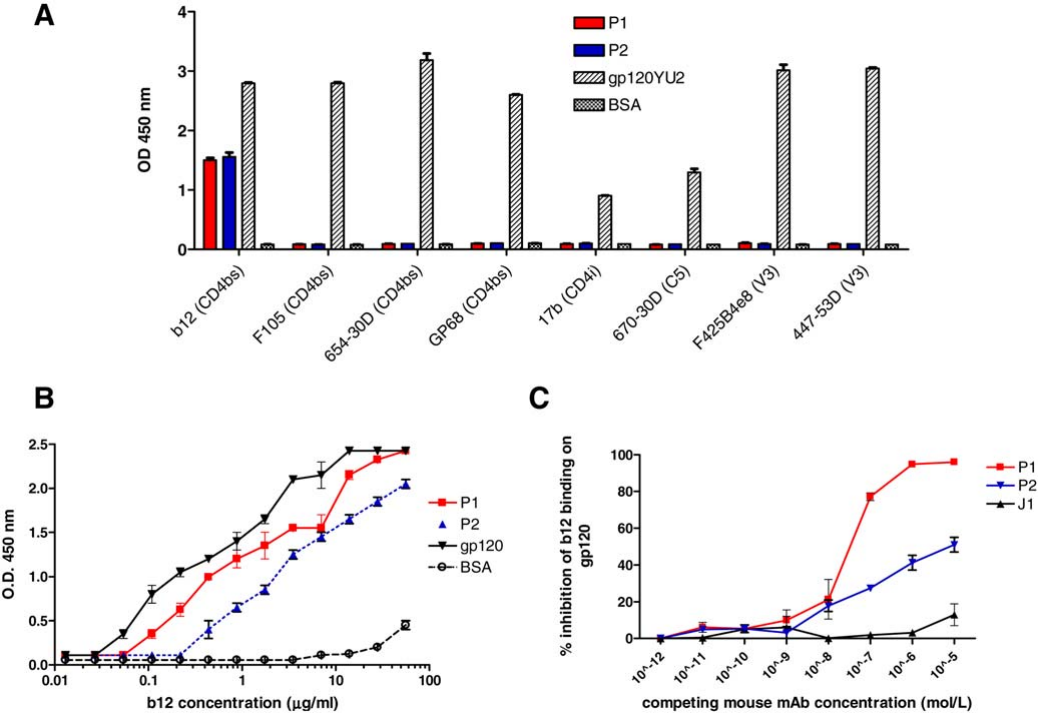
Characterization of P1 and P2 binding features. (a) Recognition of P1 and P2 putative AI molecules by anti-gp120 mAbs. ELISA wells coated with P1, P2, gp120 IIIB (YU2) as positive control, or BSA (negative control) were reacted with: I) four anti-CD4bs mAbs: b12 (1.4 µg/ml), F105 (1.5 µg/ml), 654-30D (1 µg/ml) and GP68 (1 µg/ml); II) two anti-V3 mAbs: F425 B4e8 (1.5 µg/ml) and 447-52D (1 µg/ml); III) one anti-C5 mAb: 670-30D (0.4 µg/ml) and IV) one anti-CD4 induced epitope mAb 17b (1 µg/ml). All monoclonals were obtained through the NIH AIDS Research and Reference Reagent Program, Division of AIDS, NIAID, NIH. Interestingly, P1 and P2 were specifically recognized only by b12, the most potent mAb used in this study in terms of neutralizing activity on primary isolates. The AI molecules were also tested with different preparations of standard IgG and with all serum fractions discarded during the purification process of anti-CD4bs IgG, giving in all cases a negative result (data not shown). (b) Titration of b12 on P1, P2, gp120 and BSA. (c) Competition ELISA. The ability of P1 and P2 to inhibit the binding of b12 to immobilized gp120 was tested in a competition ELISA. 96-well flat bottom plate wells were coated overnight at 4°C with HIV-1 YU2 gp120 (100 ng/well) and then blocked with 1% BSA-PBS. b12 (1 µg/ml) and serial dilutions (from 10^−5^ M to 10^−12^ M) of P1, P2 and J1 control mAb were added to the wells. b12 residual binding was detected with enzyme-linked goat anti-human IgG.

To further characterize their binding specificity, P1 and P2 AI clones were also tested (as Fab fragments) using a panel of anti-gp120 human monoclonal antibodies (mAbs) directed against crucial HIV-1 epitopes. In this assay, 100 ng each of the P1 and P2 Fab were used as antigen in ELISA wells, and anti-gp120 mAbs were tested for reactivity with P1 and P2. Recombinant gp120 (YU2 strain) and BSA were used as positive and negative controls respectively. Importantly, P1 and P2 mAbs were recognized by mAb b12, a broadly neutralizing human monoclonal antibody directed against the gp120 CD4bs [27]–[30] (Figure 1A and 1B). Of note, the non neutralizing anti-CD4bs mAb, F105, did not recognize P1 and P2. Additionally, P1 and P2 were tested in a competition ELISA for the ability to inhibit the binding of b12 to immobilized gp120. Both AI mAbs were able to inhibit this binding; in particular P1, at the highest concentration used, was able to completely inhibit the binding of b12 to gp120 (Figure 1C). P1 and P2 did not inhibit the binding to gp120 of any of the other anti-gp120 mAbs used in this study (data not shown).

**Figure 2:**
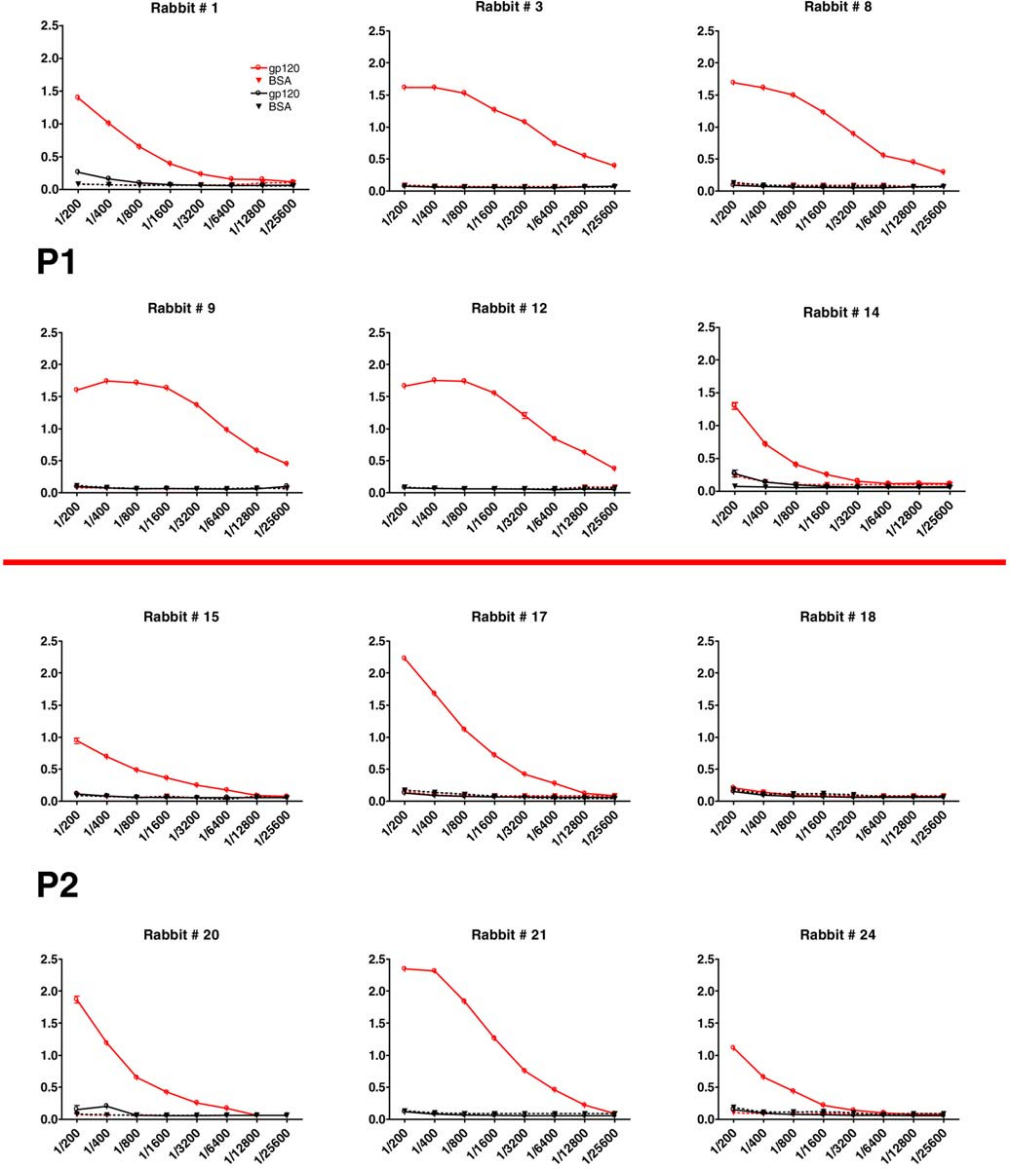
Evaluation of P1 and P2-induced antibody response in rabbits and of its neutralizing activity in an Env-based pseudovirus assay. ELISA titers of preimmune (black) and immune (red) rabbits' sera on gp120 IIIB (continuous line) and BSA (dotted line) after three immunizations with the reported antigens. Data regarding the gp120 (positive control) and the J1 (negative control) groups are not reported, but for all animals the titers were >1∶12800 and <1∶25, respectively. Moreover, among the animals immunized with the mouse mAbs, no difference was evidenced in terms of response elicited against the specific molecule used for the immunizations (data not shown).

To confirm the AI nature of P1 and P2, the mAbs were used as Fab fragments to immunize 5-week-old *New Zealand* female rabbits. In particular, 4 groups of 6 rabbits each were immunized, with P1, P2, an irrelevant mouse mAb (J1; negative control), or monomeric gp120 IIIB (positive control). The animals were immunized biweekly with 200 µg of each antigen, suspended in complete Freund's adjuvant (priming) or in incomplete Freund's adjuvant (two following boosts). After this schedule, the preimmune and immune sera of each animal were titered in ELISA using monomeric gp120. All of the P1-receiving rabbits featured a strong anti-gp120 response, with titers ranging from 1∶1600 to 1∶25600; 5 out of the 6 P2-immunized rabbits showed a less intense, albeit detectable, anti-gp120 response with titers ranging from 1∶800 to 1∶6400. None of the J1-immunized control rabbits showed any anti-gp120 response (Figure 2).

**Figure 3:**
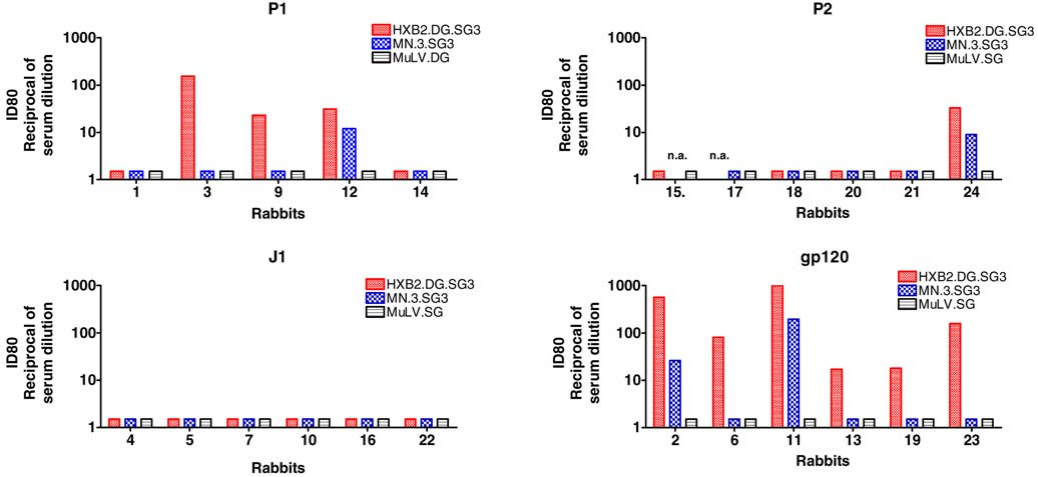
Evaluation of P1 and P2-induced neutralizing activity in an Env-based pseudovirus assay. Data are reported as reciprocal of serum dilutions giving 80% of pseudovirus neutralization (ID80). Neutralization data on Murine Leukemia Virus (MuLV) are also reported as control. For sample shortage due to technical problems it was not possible to perform the assay with sera from rabbit # 8 (P1 group), and for rabbits # 15 and # 17 (P2 group) on MN and HXB2 pseudoviruses, respectively.

To evaluate whether the anti-gp120 positive rabbit sera had HIV-1 neutralizing activity, the sera were tested using an Env pseudovirus-based neutralization assay [18], [39] against recombinant viruses expressing the surface glycoproteins of two tier 1 HIV-1 strains: HXB2 and MN.3. In this triage study only tier 1 viruses were tested in order to evaluate even a minimal amount of elicited neutralizing antibodies. None of the negative control sera featured any neutralizing activity in terms of ID80 (reciprocal serum dilution giving 80% neutralization) (Fig. 2b). Neutralizing activity was demonstrated in 3 out of 5 rabbits in the P1 group (samples from 1 of the rabbits were not available), and in one rabbit of the P2 group; the neutralizing titers ranged from 1∶20 to 1∶150. Importantly, sera from 2 animals (one in the P1 and one in the P2 group) featured a neutralizing activity against both pseudoviruses (Figure 3).

Overall, we identified 2 putative CD4bs-mimicking AI mAbs specifically recognizing a cross-reacting IgG polyclonal preparation purified from the sera of a cohort of HIV-1-infected LTNP patients. Both mAbs were shown to react with the anti-CD4bs human neutralizing mAb b12, and to elicit antibodies specifically directed against gp120. Although the two pseudovirus strains tested in this study represent tier 1 viruses and much more work is certainly needed before neutralization of tier 2 viruses may be achieved, the reported data indicate that an anti-HIV-1 epitope-based vaccine approach is possible. In addition, the present study unambiguously documents that immunogens designed on the idiotype of broadly neutralizing mAbs, if derived in the proper way, could strongly help in the design of future anti-HIV-1 vaccines.

## Materials and Methods

### Purification of CD4-binding site-directed anti-gp120 IgG from *long-term non-progressor patients*

All sera were derived from *long-term non-progressor* patients chosen according to well defined criteria [16]. Each patient gave written consent to use of the sera for research purposes, and the protocol was approved by the San Raffaele Hospital's ethics committee. All different serum aliquots were pooled and the IgG fraction purified by affinity chromatography. In brief, the pooled sera were diluted with 10 volumes of phosphate buffered saline (PBS), and filtered through 0.45 µm filters (Millipore Corporation; Bedford, MA). The solution was then passed through a protein G-containing sepharose column (Gammabind sepharose™, GE Healthcare Life Sciences, UK). After a wash with 10 volumes of PBS, the bound IgG fraction was eluted with 10 volumes of 0.1 M citric acid elution buffer (pH 3), and rapidly neutralized with 1.5 M Tris-base.

Total anti-gp120 antibodies were then purified by affinity chromatography using a gp120-Sepharose column. More in details, 700 µg of a recombinant gp120 IIIB protein (ImmunoDiagnostics Inc., Woburn, MA) diluted in 4 ml of *coupling buffer* (100 mM NaHCO_3_; 500 mM NaCl; pH 8.2) were covalently coupled to 4 ml of a CNBr-activated Sepharose resin (GE Healthcare Life Sciences, UK). The solution was incubated overnight at 4°C, and the unbound antigen was eliminated by washing the resin with the *coupling buffer*. The resin was then packed in a column, and washed with 10 volumes of 10 mM Tris (pH 7.5). Further washings were subsequently performed with 10 volumes of 100 mM glycine (pH 2.5), 10 volumes of 10 mM Tris (pH 8.8) and 10 volumes of 100 mM triethylamine (pH 11.2). The column was finally equilibrated to pH 7.5 with 10 mM Tris (pH 7.5). The purified serum antibodies were diluted 1∶3 with 10 mM Tris (pH 7.5), and then consecutively passed for seven times through the column at 4°C. The column was then washed with 10 volumes of 10 mM Tris (pH 7.5), and the bound antibodies were finally eluted with 10 mM glycine (pH 2.5). All eluted fractions were then tested in ELISA, and those strongly reacting against gp120 (O.D. 450 nm >2.0) were pooled.

In order to purify the antibodies putatively directed against the CD4 binding-site (CD4bs) on gp120, the gp120 IIIB-sepharose column was saturated with an excess of soluble CD4. The anti-gp120 immunoglobulins, purified as described above, were then passed through the column, and the flow-through was collected. The flow-through fraction was then passed through another gp120-Sepharose column, prepared exactly as described above, but containing a recombinant gp120 MN. The bound fraction was eluted as described above, and used for Fab fragments production with a commercial kit (ImmunoPure Fab Preparation Kit - Pierce, Rockford, IL), following manufacturer's instructions.

### BALB/c mice immunization, hybridoma generation and selection of antibodies with putative anti-idiotype features

After approval of all parts of the project involving animals by the San Raffaele Hospital's Institutional Animal Care and Use Committee (I.A.C.U.C.), 5-week-old female BALB/c mice were immunized intraperitoneally with 50 µg of purified anti-CD4bs Fabs, resuspended in 0.5 ml of PBS and in 0.5 ml of complete Freund's adjuvant for the priming, and 0,5 ml of incomplete Freund's adjuvant for the subsequent boosts. A biweekly schedule was followed for a total of 5 administrations. Three days after the last boost the mice were sacrificed, and the spleen removed under sterile conditions. The spleen cells were separated from the fibrous component, and subsequently washed and resuspended in EMEM (Invitrogen) added with penicillin (100 mIU/ml) and streptomycin (100 µg/ml). As fusion partners for hybridoma generation, NS-1 myelomatous cells (ECACC # 85011427) were cultured in RPMI-1640 (Gibco) added with 20% decomplemented bovine foetal serum (BFS) (Gibco). The fusion was performed resuspending the spleen cells and the NS-1 cells in 1 ml of a *fusion medium* (0.5 ml of EMEM, 0.025 dimethylsulfoxide and 0.475 ml of PEG1540). After 3 minutes, 5 ml of EMEM were added to the fusion medium; after additional 3 minutes 7 ml of 20% decomplemented BFS-RPMI were added to the medium. The cells were then centrifuged (1200 rpm for 15'), and subsequently resuspended in 200 ml of HAT selective medium (150.2 ml of RPMI-1640, 40 ml of decomplemented BFS, 5.4 ml of a 7% sodium bicarbonate solution, 135 µg/ml hypoxanthine, 40 µg/ml thymidine, 2 µg/ml aminopterin). The HAT selective medium was also added with penicillin (100 mIU/ml), streptomycin (100 µg/ml), L-glutamine (2 mM) and Amphotericin B (100 µg/ml). The cell suspension was incubated for 1 h at 36.5°C in an atmosphere of 5% CO_2_, and subsequently 200 µl were added in each well of ten 96-well Microtiter plates (NUNC) for hybridoma growth. The plates were incubated as above, and routinely checked for hybridoma growth. The supernantant of each hybridoma-containing well was tested in ELISA against preparations of Fab fragments obtained from HIV-negative patients. All hybridomas reacting against these preparations were eliminated. Non-reacting hybridomas were tested for reactivity against the preparation of Fab fragments used for the immunization of mice. With this approach 2 Fabs, named P1 and P2, specifically reacting on the anti-CD4bs fraction were evidenced.

Fab fragments were obtained for P1 and P2 by cloning the genes coding for the light chain and the Fd fragment of the heavy chain into an expression vector named pRBCaf [40]. Briefly, the total mRNA was extracted from cultured cells and genes coding for the light chain and Fd fragment were amplified by RT-PCR, as previously described (CSH press, Phage display manual, ed. D.R.Burton, pag. A1.10). The amplified Fd gene was digested with *Xho*I and *Spe*I (Roche) and then cloned into pRBCaf previously digested with the same restriction enzymes. Similarly, the amplified light chain gene was digested with *Sac*I and *Xba*I (Roche) and cloned into the pRBCaf light chain cloning site previously digested with the same restriction enzymes.

Electrocompetent *E. coli* XL1-Blue (Stratagene) were then transformed by electroporation with the vectors pRBCaf-P1 and pRBCaf-P2, obtained as described above. Bacterial cultures from transformed clones were used to produce P1 and P2 through overnight induction with IPTG [isopropilβ-D-tiogalattopiranoside (Sigma), final concentration 1 mM], and subsequent cell lysis by freezing/thawing cycles. Sonicated bacterial cultures were centrifuged and the supernatant recovered and tested in ELISA for binding to human anti-CD4bs Fab fragments.

### P1 and P2 Fab fragments purification

P1 and P2 Fab were purified by immune affinity chromatography through a protein G sepharose column (Gammabind sepharoseTM, GE Healthcare Life Sciences, UK) covalently bound to goat-anti-mouse-(Fab)_2_ polyclonal IgG (PIERCE, Illinois). Briefly, bacterial cultures were induced with IPTG (final concentration 1 mM) and, after over night growth at 30°C, were centrifuged, the pellet resuspended in PBS and then sonicated. Disrupted bacteria were ultracentrifuged, the supernatant filtered and passed through the sepharose column. The column was washed with 30 ml of PBS, and the Fabs were eluted with 10 mL of elution buffer (H_2_O/ HCl pH 2.2). The eluate was collected as 2 ml fractions quickly neutralized with a basic solution (Tris-base 1 M, pH 9). The absorbance of each fraction was measured at 280 nm in order to evaluate the amount of purified Fab. The purity of the purified protein was evaluated through SDS-PAGE. Purified Fab were tested in ELISA for binding to human anti-CD4bs Fab.

### Immunization of rabbits with P1 and P2

In order to evaluate the ability of the mouse AIs to induce an anti-HIV-1/gp120 specific response, purified P1 and P2 were used to immunize 5-week-old New Zealand female rabbits. In particular, 4 groups of 6 rabbits each were formed (group A: animals immunized with P1; group B: animal immunized with P2; group C: animals immunized with an irrelevant mouse mAb (J1) used as negative control; group D: animals immunized with monomeric gp120 used as positive control). The animals were immunized biweekly with 200 µg of each antigen, resuspended in complete Freund's adjuvant (Pierce, Rockford, IL) (priming) or in incomplete Freund's adjuvant (Pierce, Rockford, IL) (two following boosts). The sera collected before and after this immunization schedule were titered in ELISA using monomeric gp120 YU2.

### Virus neutralization assays

HIV-1 Env pseudoviruses were prepared by cotransfecting 293T cells with an Env expression plasmid containing a full gp160 gene and an *env*-deficient HIV-1 backbone vector (pSG3ΔEnv), as previously described [41]. Virus-containing culture supernatants were harvested 2 days after transfection, centrifuged, filtered through 0.45-µm filter and stored at −80°C. We used two Env-pseudoviruses (HxB2 and MN) to assess the neutralization activity of the rabbit sera. Envelope glycoproteins from murine leukaemia virus (MuLV) were used as negative control envelope. Pseudovirus neutralization was measured as a function of Tat-induced luciferase reporter gene expression after a single round of infection in TZM-bl cells, as previously described [18], [39]. TZM-bl cells were obtained from the NIH AIDS Research and Reference Reagent Program. These cells express CD4, CXCR4 and CCR5 and contain an integrated reporter gene for firefly luciferase under the control of HIV-1 LTR. The level of viral infection was quantified by measurement of relative luciferase units (RLU) that are directly proportional to the amount of virus inputs. Briefly, 40 µl of virus was incubated for 30 min at 37°C with serial dilutions of test serum samples (10 µl) in duplicate wells of a 96-well flat bottom culture plate. The final serum dilution was defined at the point of incubation with virus supernatant. 10.000 TZM-bl cells were then added to each well in a total volume of 20 µl and plates were incubated overnight at 37°C in a 5% CO2 incubator. One set of eight wells received mock antibody followed by virus and cells (controls wells for virus entry) and a set of eight wells received cells with mock virus (to control for luciferase background). Viral input was set at a multiplicity of infection (MOI) that resulted in 100.000–40.000 RLU. After over night incubation, 150 µl of fresh medium was added to each well and incubated for 24 h at 37°C in a 5% CO2 incubator. To determine RLU, cell culture medium was aspirated from wells followed by addition of 50 µl of cell lysis buffer (Promega, Madison, WI). 30 µl of cell lysate was transferred to wells of a black Optiplate (Perkin–Elmer) for measurement of luminescence using a Perkin–Elmer Victor-light luminometer that injects 50 µl of luciferase substrate reagent to each well just prior to reading RLU. To determine the serum inhibitory dilution that resulted in a 80% reduction in RLU, serum neutralization curves were fit by non-linear regression using a four-parameter hill slope equation programmed into JMP statistical software (JMP 5.1, SAS Institute Inc., Cary, NC). The results are reported as the serum neutralization ID80, which is the reciprocal of the serum dilution producing 80% virus neutralization.
